# A Case Report of Ruptured Intracranial Dermoid Cyst

**DOI:** 10.7759/cureus.51718

**Published:** 2024-01-05

**Authors:** Sharif Alfeki, Abdullah Alsaedi, Osama Alsheikh, Abdullah AlSafar, Atheer Alqubaysi, Amjad Aljubairy, Feras Ageeli, Mazen Ali

**Affiliations:** 1 Emergency Medicine, Dallah Hospital, Riyadh, SAU; 2 General Practice, King Abdulaziz University, Jeddah, SAU; 3 General Practice, King Khalid University, Abha, SAU; 4 General Practice, Ibn Sina National College for Medical Studies, Jeddah, SAU; 5 General Practice, Jazan University, Jazan, SAU

**Keywords:** congenital lesions, computed tomography scan, meningitis, cyst rupture, dermoid cyst, severe headache

## Abstract

Intracranial dermoid cysts, rare congenital lesions originating from ectodermal elements during neural tube closure, are explored in the context of a 45-year-old female presenting with a sudden-onset severe headache, nausea, and vomiting. A thorough neurological examination revealed no focal deficits, prompting a computed tomography scan that identified multiple extra-axial intracranial fat density lesions indicative of dermoid cysts. Laboratory and cerebrospinal fluid analysis confirmed inflammatory changes, characterized by an increased white blood cell count. Successful surgical intervention followed, resulting in the complete removal of the cyst and the patient's subsequent full recovery with the resolution of symptoms. This case highlights the intricate nature of intracranial dermoid cysts and underscores the critical importance of prompt recognition in effectively mitigating potential complications.

## Introduction

Intracranial dermoid cysts are rare congenital lesions, representing less than 1% of all intracranial tumors [1,2]. These cysts arise from the inclusion of ectodermal elements during neural tube closure, typically during the third to fifth weeks of gestation. Composed of stratified squamous epithelium, sebaceous glands, and hair follicles, dermoid cysts manifest as encapsulated lesions within the central nervous system [2]. While often discovered incidentally, they may present with a spectrum of symptoms, including headaches, seizures, and neurological deficits, depending on their size, location, and potential for rupture [2]. This case report centers on a 45-year-old female who presented to the emergency department with a severe headache and was subsequently diagnosed with a ruptured intracranial dermoid cyst. Through a detailed exploration of the clinical presentation, diagnostic workup, and subsequent surgical intervention, this case provides insights into intracranial dermoid cysts.

## Case presentation

A 45-year-old female presented to the emergency department with a sudden onset of severe headache, accompanied by nausea and vomiting. The patient reported a history of intermittent headaches over the past few months but denied any significant neurological symptoms. Her medical history was unremarkable, with no known chronic medical conditions, and she was not on any medications.

During the physical examination, the patient appeared distressed and complained of a persistent throbbing headache localized to the right frontal region. Neurological examination revealed no focal deficits, and the patient's vital signs were within normal limits. However, the severity and persistence of the headache raised concerns, prompting a non-contrast computed tomography scan of the head.

**Figure 1:**
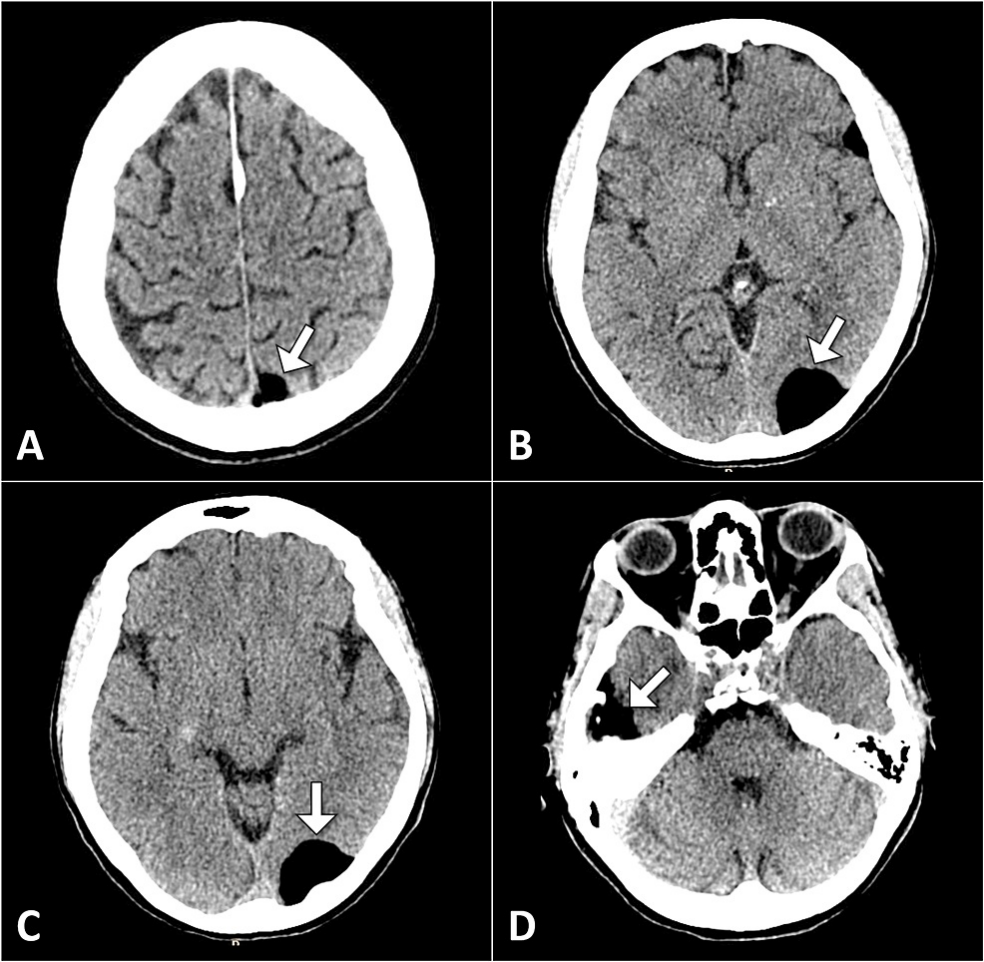
Multiple axial head CT images illustrating extra-axial fat density lesions (indicated by arrows), indicative of a dermoid cyst. CT: computed tomography.

The computed tomography scan revealed multiple extra-axial lesions in the left parasagittal region and the right temporal region. These lesions had a homogeneous fat density with no calcification or soft tissue components. The imaging findings were suggestive of a ruptured dermoid cyst (Figure 1).

In addition to the clinical and radiological aspects of the case, the patient's initial complete blood count and basic metabolic panel revealed normal ranges, with a white blood cell count of 8.5 × 10^9^ cells/L and unremarkable electrolyte levels, suggesting an absence of systemic abnormalities. Notably, cerebrospinal fluid analysis revealed an aseptic inflammatory response, characterized by an elevated white blood cell count of 150 cells/μL (normal range: 0-5 cells/μL) and increased protein levels of 80 mg/dL (normal range: 15-45 mg/dL). These findings were consistent with the diagnosis of ruptured intracranial dermoid cysts (Table 1).

**Table 1:** Initial laboratory investigations.

Laboratory test		Patient's value	Normal range	Indicator
Complete blood count	White blood cell count	8.5 × 10^9^ cells/L	4.0–11.0 × 10^9^ cells/L	Normal
	Hemoglobin	12 g/dL	12–16 g/dL	Normal
	Platelets	250 × 10^3^/μL	150–450 × 10^3^/μL	Normal
Complete metabolic panel	Sodium	138 mmol/L	135–145 mmol/L	Normal
	Potassium	4.2 mmol/L	3.5–5.0 mmol/L	Normal
	Blood urea nitrogen	12 mg/dL	7–20 mg/dL	Normal
	Creatinine	0.9 mg/dL	0.6–1.1 mg/dL	Normal
	Glucose	90 mg/dL	70–100 mg/dL	Normal
Cerebrospinal fluid analysis	Protein levels	80 mg/dL	15–45 mg/dL	High
	White blood cell count	150 cells/μL	0–5 cells/μL	High
	Glucose levels	60 mg/dL	40–70 mg/dL	Normal

The patient was promptly admitted for further management. A neurosurgical consultation was obtained, and a decision was made to proceed with surgical intervention based on the imaging findings. The surgical team successfully resected the dermoid cyst, taking care to address the spilled contents and minimize the risk of infection. Intraoperatively, the cystic lesion was found to contain hair, sebaceous material, and desquamated epithelial cells, confirming the diagnosis.

The postoperative hospital course was uneventful, with the patient experiencing a gradual resolution of her headache. She was monitored closely for any signs of infection or neurological deterioration. Histopathological examination of the excised specimen confirmed the diagnosis of a dermoid cyst. Following discharge, the patient was scheduled for regular follow-up appointments to assess her neurological status and monitor for any potential complications.

## Discussion

Intracranial dermoid cysts are slow-growing, benign tumors that originate from ectodermal cell rests. Though these cysts are generally asymptomatic and discovered incidentally, rupture can lead to a spectrum of neurological symptoms and complications. Ruptured intracranial dermoid cysts often present with a diverse array of symptoms, depending on the location and extent of the rupture. Common clinical manifestations include headaches, nausea, vomiting, and signs of increased intracranial pressure. Neurological deficits, such as cranial nerve palsies and focal neurological signs, may also be observed [1-3].

Diagnostic imaging plays a pivotal role in the assessment of ruptured intracranial dermoid cysts. A computed tomography scan reveals extra-axial intracranial fat density lesions, as in our case. Magnetic resonance imaging is the modality of choice, providing detailed information on cyst location, size, and content. Ruptured cysts may exhibit a unique radiological appearance, including lipid droplets in the subarachnoid space and a characteristic hyperintense signal on T1-weighted imaging [2,3].

The pathogenesis of intracranial dermoid cyst rupture remains a subject of ongoing research. Mechanical factors, trauma, or spontaneous rupture may trigger leakage of cyst contents into the surrounding cerebrospinal fluid, leading to inflammatory reactions and neurological symptoms [3,4]. This explains the cerebrospinal fluid analysis in our case in the presence of a high white blood count.

Rupture of intracranial dermoid cysts can result in various complications, including chemical meningitis, aseptic meningitis, and vasospasm [2-4]. Additionally, the release of lipid-rich material into the subarachnoid space may lead to granulomatous reactions, further exacerbating neurological symptoms and complicating the clinical course. The management of ruptured intracranial dermoid cysts requires a multidisciplinary approach [4,5]. Surgical intervention is often considered in symptomatic cases, with the goal of cyst removal and prevention of recurrence. However, the timing and extent of surgery must be carefully evaluated, considering individual patient factors and the potential risks associated with the procedure.

## Conclusions

In conclusion, the presented case of a ruptured intracranial dermoid cyst serves as a noteworthy illustration of the complex nature of these rare congenital lesions. The patient's clinical journey, marked by a sudden-onset severe headache and subsequent neurosurgical intervention, highlights the paramount importance of prompt recognition and decisive management in mitigating potential complications associated with cyst rupture. The successful surgical outcome, characterized by the meticulous resection of the dermoid cyst and subsequent resolution of symptoms, underscores the effectiveness of a multidisciplinary approach involving clinical evaluation, advanced imaging, and surgical expertise.
